# Stepwise Reduction of Dietary Phosphorus in Diets for Piglets and Fattening Pigs of Different Genetic Origin Housed under Various Station Environments—A Ringtest

**DOI:** 10.3390/ani13111774

**Published:** 2023-05-26

**Authors:** Jochen Krieg, Gerhard Stalljohann, Michael Oster, Ralf Pfuhl, Bernd Reckels, Wolfgang Preissinger, Manfred Weber, Andrea Meyer, Dieter Feuerstein, Stephan Schneider

**Affiliations:** 1 Landwirtschaftskammer Nordrhein-Westfalen (LWK NRW), 59505 Bad Sassendorf, Germany; 2 Research Institute for Farm Animal Biology (FBN), 18196 Dummerstorf, Germany; 3 Institute for Animal Nutrition, University of Veterinary Medicine Hanover, Foundation, 30173 Hannover, Germany; 4 Bayerische Landesanstalt für Landwirtschaft (LfL Bayern), 97359 Schwarzach am Main, Germany; 5 Landesanstalt für Landwirtschaft und Gartenbau (LLG) Sachsen-Anhalt, 39606 Iden, Germany; 6 Landwirtschaftskammer Niedersachsen, 30453 Hannover, Germany; 7 BASF SE, 67063 Ludwigshafen am Rhein, Germany; 8 Bayerische Landesanstalt für Landwirtschaft (LfL Bayern), 85586 Poing, Germany

**Keywords:** pig nutrition, phytase, mineral phosphorus intake, fattening pigs, piglets, sus scrofa

## Abstract

**Simple Summary:**

To reduce the impact of livestock on the environment, in modern rations for farm animals the concentration of relevant nutrients is aimed to be lowered. The main nutrients in focus are nitrogen and phosphorus. Because phosphorus is essential for bone development, the degree to which dietary phosphorus can be decreased is limited. There are case reports that pigs indicate leg problems under specific practical conditions, and theories that this might be linked to reduced phosphorus level in feeds are circulating. To verify whether these observations are connected to the nutritionally recommended reduction of dietary mineral phosphorus and to increase the acceptance of these feeding regimes on farmer level, three diets with variable phosphorus levels were fed throughout grower and finisher stages. Performance, bone mineralization, blood parameters and selected mineral transporters involved in P homoeostasis were studied. The results did not show any indication of an insufficient outcome. Thus, it is concluded that the digestible phosphorus supply was sufficient within the range of this study. It is further highlighted that it is essential to decrease the calcium concentration in phosphorus-reduced diets to maintain high P digestibility and availability, which is often neglected under practical conditions.

**Abstract:**

The reduction of emissions of nutrients from livestock is one of the main topics in areas with intensive animal husbandry. In order to minimize the loss of nutrients into the environment, it is common practice to feed animals as close as possible to metabolic demands. For phosphorus (P), there are various studies for swine and poultry, which showed that a reduction of dietary P levels is possible, if a sufficient level of phytase is added to the diet. The supplementation of a sufficient dosage of phytase to plant-based diets leads to an increase in digestible phosphorus (dP) upon the hydrolisation of phytate (InsP_6_) to P and lower inositol-phosphates. However, most of these studies were conducted under standardized experimental conditions. In terms of transfer to practical conditions with varying housing, management and genetics, there are concerns that have led to speculation by farmers and veterinarians whether the reduction of dietary P could negatively affect bone health and therefore animal welfare. In order to test whether a reduction of dietary P according to the recommendations for dP of the German Society of Nutrition Physiology (GfE) affects bone mineralization and growth performance, a ringtest was conducted where piglets and fattening pigs were fed at four experimental stations with three centrally produced diets from the same batches. The diets contained three different levels of P and were designed to reflect practical diets. The P level decreased from diet one to three, respectively. Diets one and two were calculated to contain P levels, which are typically fed under practical conditions in Germany. The third diet was optimized to fulfill the requirements of dP by the GfE. The animals were fed in two phases as post-weaning piglets (8–15 kg and 15–28 kg BW) followed by a three-phase fattening regime (28–60 kg, 60–90 kg and 90–120 kg BW). Individual body weight and feed consumption (pen basis or individually, depending on the experimental station) were recorded for every feeding phase. At the end of the experiment, animals were slaughtered. At one experimental station, additional blood serum, metatarsi of the left leg and kidney tissue were sampled to analyze serum P concentration, expression of P transporters in the kidney and bone traits. In two experimental stations, femur and vertebra were sampled, and bone ash was determined. Overall, animal performance and all other traits analyzed did not differ between the treatment with the highest and the treatment with the lowest dietary P concentration. The results demonstrate that it is possible to decrease dietary P according to the recommendations for dP of the GfE, without impairing the animals’ performance or mineral homeostasis and health. A reduction of total P by reducing mineral P to the levels of the present study require the supplementation of phytase to achieve sufficient concentrations of dP.

## 1. Introduction

It is acknowledged that an excess of phosphorus (P) in feeds of farm animals leads to an increase in P excretion and therefore increases the environmental burden of animal husbandry. Hence, it is necessary to provide P as close to the animal requirements as possible, without risking negative effects on the health or the performance of the animals [1]. Various studies have addressed effects of a variable dietary P supplementation on performance and bone development in pigs (e.g., [2,3,4]) and poultry [5,6]. Under practical conditions, diets include a safety margin to avoid possible negative effects of a non-sufficient P supplementation [7]. However, high safety margins far beyond the requirement lead to excess P supplementation, thus increasing the P excretion and promoting eutrophication of water bodies [8,9,10]. The wide use of phytases in today’s non-ruminant diets allows a strong reduction in dietary P concentration, while the level of available P still meets the requirement of the animal [11,12]. This effect is caused by an increase in P digestibility upon phytate hydrolysis by phytases [13]. This effect allows a reduction of dietary P, without reducing the P available for the animal. While the magnitude of the effects of phytase supplementation on P digestibility and availability can vary between products, the incremental effect of phytase supplementation on P digestibility in pigs is well studied (e.g., [14]) and can be considered as generally accepted. From an environmental point of view, the reduction of P input into water bodies is mandatory and is thus included in various laws and guidelines. The German Agricultural Society (DLG), for example, has defined standard feeding strategies to limit the emissions and thus the environmental impact of animal husbandry [15,16]. However, there are major concerns by farmers and veterinarians that the reduced P-concentration in today´s practical diets in combination with the high weight gain of modern breeds might impair bone strength and animal health. Because the feeding strategies described by the DLG are designed to supply nutrients above the recommendations of the German Society of Nutrition Physiology (GfE) [17], a need for intensive interdisciplinary research becomes essential. Compared to international recommendations, e.g., those by the NRC [18], the FEDNA [19] or the Brazilian recommendations (Universidade Federal de Viçosa-Departamento de Zootecnia [20]), the concentrations of P/dP stated by the GfE [17] appear to be adequate to meet the requirements of piglets and fattening pigs.

This study was conducted to test whether a diet formulation based on the digestible P (dP), according the GfE recommendations, offers sufficient P supply for piglets and fattening pigs for both high performance and bone mineralization. Additionally, a third treatment, which reflects a feeding strategy that can be found under practical conditions in Germany, was tested. Animals were housed at three and four experimental stations for piglets and fattening pigs, respectively. Diets were centrally produced and offered for ad libitum consumption from weaning to slaughter at 120 kg body weight. The hypothesis was that a reduction of dietary P is possible with high performance modern breeds and does not impair performance or bone mineralization, if the recommendations of the GfE for dP are met.

## 2. Materials and Methods

### 2.1. Animals and Housing

The experimental stations that were part of this ringtest were located in Germany in North Rhine Westphalia (experimental station 1), Saxony-Anhalt (experimental station 2), Bavaria (experimental station 3) and Lower Saxony (experimental station 4). All housing conditions were in accordance with the German animal welfare legislation and approved by the respective animal welfare commissioner. The number of repetitions and whether the data were collected for individual animals or on a pen basis is summarized in Table 1. The used genetics differed between the experimental stations: At stations 1 and 4, crosses of Pietrain (PIC408) and either Topigs40, Topigs70 or db.Viktoria were used. At station 2, Pietrain x Topigs were used. Station 3 fed animals of Pietrain x (DL x DE). Due to the different housing conditions and slaughter facilities, the number of repetitions used for biological data and tissue samples differed between the stations.

### 2.2. Feed Formulation and Analyses

Diets were calculated to meet or exceed the requirements of the animals [17] and to be isocaloric and identical in all nutrients except P and Ca. In order to avoid the influence of different feedstuffs, the components between the treatments within one phase were identical. The P concentration in diets 2 and 3 was achieved by substitution of mineral P by barley. In order to achieve similar Ca:dP ratios in all treatments, calcium was added as CaCO_3_ (Table 2 and Table 3) and reduced in P-reduced diets.

Ground feed samples were analyzed for crude nutrients, starch, sugars, amino acids, Ca and P according to the certified methods (Verband Deutscher Landwirtschaftlicher Untersuchungs- und Forschungsanstalten, VDLUFA, methods 4.1.1, 5.1.1, 6.1.1, 7.1.1, 7.2.1, 8.1, 4.11.1, 4.11.2, 10.8.2, 10.6.3). Neutral detergent fiber after amylase treatment excluding residual ash (aNDFom) and acid detergent fiber excluding residual ash (ADFom) were determined according to methods 6.5.1 and 6.5.2 (VDLUFA). Phytase activity was analyzed by BASF SE according to ISO EN 30024.

### 2.3. Measured Performance Traits

The animals were individually weighed at weaning, which was also the start of the experimental phase, and after every feeding period. The feed consumption was recorded on a pen basis and, if possible, individually (Table 1). The animals remained in the respective treatments in all phases. The number of repetitions for all feed-related performance traits (over all stations) was n = 46 in the piglet phases and n = 194 in the fattening phases. All weight data were recorded on animal basis, which resulted in n = 695 animals/repetitions for the piglet phases and n = 582 animals/repetitions for the fattening phases over all experimental stations. The number of animals in the fattening phases was lower than in the piglet phases due to limitations in the experimental stations and because some of the animals were slaughtered at 28 kg. At the end of the experiment, the animals were slaughtered in commercial slaughter facilities, specific for every experimental station, or at an experimental slaughter facility at experimental station 3. The carcass, except the sampled tissues, was supplied for human consumption.

### 2.4. Expression Analyses of Na/P Co-Transporters in the Kidney

Cortex tissue of the left kidneys was obtained from animals in experimental station 3 and stored at −80 °C until further analysis. Total RNA of the kidney samples (n = 40) was isolated using TRI reagent according to the manufacturer’s instructions (Sigma-Aldrich, Taufkirchen, Germany). Samples were treated with DNaseI (Roche, Mannheim, Germany) and purified with a column-based RNA extraction kit according to the manufacturer’s protocols (NucleoSpin RNAII, Macherey-Nagel, Düren, Germany). The amount and purity of the replicates were checked using a NanoDrop ND-2000 photospectrometer (NanoDrop, Peqlab, Erlangen, Germany). All RNA samples were stored at −80 °C until further analyses.

First-strand cDNA synthesis was performed in a reaction with random primers, oligo d(T) 13VN, Superscript III reverse transcriptase (Invitrogen, Karlsruhe, Germany), and RNAsinPlus RNase inhibitor (Promega, Heidelberg, Germany). The cDNA samples were stored diluted in distilled water at −20 °C until further analysis. Transcript levels of selected target (Na-P-cotransporter) and reference genes (*RPL10*, *RPL32*) were quantified by RT-qPCR as previously described [21]. Individual mRNA samples (n = 10/treatment) were analyzed in duplicate on a LightCycler 480 system using the LightCycler 480 SYBR Green I Master (Roche, Mannheim, Germany) according to the manufacturer’s instructions. After an initial denaturation step at 95 °C for 10 min, 40 PCR cycles were performed, including denaturation at 95 °C for 15 s, annealing for 10 s, and extension/fluorescence uptake at 72 °C for 15 s. The absence of non-specific products was verified by melting curve analysis and gel electrophoresis. Measured Ct values were converted to copy numbers using a standard curve generated by amplification of serial dilutions of an appropriate reference amplicon (10^7^–10^2^ copies). Analyses comprised all nine currently annotated Na-P-cotransporters in pigs, i.e., *SLC17A1* (NPT1), *SLC17A2* (NPT3), *SLC17A3* (NPT4), *SLC17A4* (NPT5), *SLC20A1* (PiT1), *SLC20A2* (PiT2), *SLC34A1* (NPT2a), *SLC34A2* (NPT2b), *SLC34A3* (NPT2c).

### 2.5. Minerals in Blood Serum

Trunk blood samples were obtained from slaughter at station 3. Serum was retrieved via centrifugation (3500 g for 10 min). Serum levels of calcium (n = 40) and inorganic P (n = 40) were determined using commercial colorimetric assays on the Fuji DriChem 4000i platform (FujiFilm, Minato, Japan).

### 2.6. Bone Mineralization

Vertebral bodies were sampled from the 13th/14th rib arches (experimental station 2: n = 40; experimental station 3: n = 24). Furthermore, femurs were taken from the left half of the body (experimental station 2: n = 40; experimental station 3: n = 36). A 2 cm section was taken from the proximal femoral epiphysis and stored at −20 °C until further analysis. Ashing of the samples took place for 7 h at 600 °C in a muffle furnace. After cooling, the samples were digested with 0.5 mL of a 30% hydrogen peroxide solution and ashed again (10 min at 600 °C).

Data on metatarsal mineralization was collected on 42 flesh- and tendon-free metatarsi III. The bone mass on thawed specimens was weighed on a top pan balance (determination of uS). The dry matter content of the bone pieces was determined by weighing the material before and after 48 h of lyophilization on an analytical balance. During subsequent pre-defattening, the bone pieces were defatted with petroleum ether in the Soxhlet apparatus. Both the mass of fat recovered and the mass of pre-defatted bone pieces were recorded on an analytical balance after cooling in the desiccator and the fat-free DM (ff DM) content was calculated.

### 2.7. Calculations and Statistics

The average daily weight gain (ADG), feed consumption, and feed conversion ratio (FCR) were calculated for each feeding period (2 piglet feeding periods and 3 fattening periods) and over the complete piglet period (8–28 kg BW) and fattening period (28–120 kg BW). Effects of the diets on the weight and slaughter data were evaluated using the model

(1) $$y_{i,j,k}={diet}_{i}+{station}_{j}+{sex}_{k}+({diet}_{i}\times {station}_{j})+({diet}_{i}\times {sex}_{k})\;\;\;\;\;\;\;+({station}_{j}\times {sex}_{k})+({diet}_{i}\times {station}_{j}\times {sex}_{k})+e_{i,j,k}$$

where diet describes the dietary treatment (T1, T2 or T3), station is the experimental station (1, 2, 3 and 4) and sex is the sex of the animal (female or barrow). Because the feed consumption was not recorded on an animal basis during the piglet period, data of the first two phases related to feed consumption were evaluated without the effect of the animals’ sex on a pen basis. Data on bone mineralization and tissue analyses were statically analyzed following the same model as piglet data, which did not include the animals’ sex, but each animal was used as a repetition. Results were considered significant at *p* < 0.05.

## 3. Results

### 3.1. Chemical Composition and Animal Performance

The calculated chemical compositions of the diets were confirmed by chemical analyses and only minor deviations within the analytical variation were found (Table 2 and Table 3 and Tables S1 and S2).

Mortality was not related to the dietary treatments and overall was at a low level. Data on live weight development during the piglet and fattening phases are shown in Table 4 and Table 5. As seen in Table 4, at the beginning of the feeding trial, the piglet weight was similar for all three feeding groups within one station.

The weight of piglets after feeding phase 1 was significantly lower for animals that received T2 than for animals receiving the other two treatments. The same significant effect was found at the end of piglet feeding phase 2. The extent of the reduction in BW due to T2 was stronger at experimental station 4 than at the other stations (compared to T1—station 1: −1.5 kg; station 2: −1.6 kg; station 3: −4.8 kg). As indicated by the differences in animal weight, the ADG of animals receiving T2 was significantly lower during the two piglet feeding phases. Only at experimental station 1 no difference between the ADG of animals from dietary treatment groups T2 and T3 during the second feeding phase was found. Over the complete piglet phase, the ADG was significantly reduced for animals fed treatment T2 compared to animals receiving T1 or T3 at all stations. As described for the BW, the extent that ADG was reduced at the end of the piglet phase was stronger at experimental station 3 than at the other stations (T1 vs. T3: station 1 −38 g/d; station 2 −42 g/d; station 3 −123 g/d).

Weight development of animals during the fattening phases is given in Table 5.

After the first fattening phase, animals of dietary treatment T2 were significantly lighter at experimental station 4 but not at the other stations. At the end of the experiment, the same effect was found: animals receiving diet T2 had lower BW at station 3. The animals receiving diet T1 and diet T3 had similar body weights throughout the experiment.

After the first fattening period, BW of the animals at experimental stations 1, 2 and 4 was similar between the dietary treatments. Animals at station 3 had a significantly lower BW when receiving T2. At the end of the second fattening phase, the weight of animals receiving T1 was significantly lower compared to animals receiving treatment T2 and T3 at experimental station 2. At experimental station 3, animals fed T2 were significantly lighter than animals of the other treatments. At the end of the experiment, animals receiving T2 were lighter compared to the other treatments. This effect was independent from the experimental station. As indicated by the same ranking of the treatments at the end of the experiment as that after the piglet phases (T1 = T3 > T2), ADG was not influenced by the dietary treatments. After the first fattening phase, the ADG of barrows at experimental station 1 was significantly lower when fed diet T2. At all other stations and for all genders, weight gain in the first fattening phase was similar between the treatments.

Average daily feed consumption and feed conversion ratio during the piglet and fattening phases are given in Table 6 and Table 7. The feed consumption in all piglet phases was significantly influenced by the experimental station but was similar between the dietary treatments (Table 6, station 1: 625 g/d, station 2: 837 g/d, station 3: 701 g/d).

Because ADG was influenced by the dietary treatment T2, FCR was significantly higher for animals receiving dietary treatment T2 in the first piglet phase. During the second piglet feeding phase, FCR was significantly increased by T2 in experimental station 3, but not at the other experimental stations. Over the whole piglet phase, T2 led to an increased FCR at experimental stations 2 and 3. Differences in FCR at experimental station 1 were only numerical.

During the second fattening phases, the feed consumption was decreased by T2 (Table 7, T1: 2.60 kg/d; T2: 2.57 kg/d; T3: 2.66 kg/d; *p* = 0.026).

For all other fattening phases, except that of phase 2, and over all fattening phases, the feed consumption was similar between the treatments. The FCR during the first fattening phase was lower for T2 than for T1 and T3. In the second fattening phase, FCR was not influenced at station 1 and 3, but decreased for T2 at station 2. At station 4, animals receiving T2 had a significantly higher FCR than animals receiving T3. During the third fattening phase, FCR was similar for all dietary treatments within one station. The FCR calculated over the complete fattening period was lower for animals receiving diet T2 compared to diet T1 and intermediate for T3.

### 3.2. Blood Serum Analyses and Transporter Expression

Blood serum analyses and expression data for genes involved in the renal transcellular P-transport are given in Table 8 and Table 9. The serum inorganic P and Ca concentrations did not show an effect due to the variable dietary P concentrations in experimental station 3 (Table 8).

The expression of genes that are coding for Na-P-cotransporters in the kidney cortex were not influenced by the dietary treatments (Table 9).

### 3.3. Bone Mineralization Measures

Weight, DM, ff DM, ash and Ca and P concentration of metatarsi at the end of the piglet feeding periods are given in Table 10.

At station 3, the fat-free DM was significantly lower for animals receiving T2 at the end of the piglet period. No other effect of the dietary treatment on other metatarsi measures was observed.

Femur length, weight and ash of animals at the end of the experiment from experimental stations 2 and 3 are given in Table 11.

The dietary treatment did not influence recorded characteristics of the femur. At experimental station 2, animals receiving T2 had significantly higher vertebra ash concentration than animals receiving T1 or T3 at station 2. There was no effect of the dietary treatment at experimental station 3.

## 4. Discussion

Mortality was overall at a low level and no connection between death of animals and the dietary treatment or experimental station was given. The animal performance was overall on a level typical for each experimental station or superior to the typically achieved performance. It is possible that different breeds show, e.g., due to differences in the daily weight gain, different reactions to the reduction of dietary P. As the stations 1–3 used different breeds of pigs, the factor “pig breed” cannot be separated from environmental effects in the current study and is therefore included in the station effect. Hence, the station effect can be seen as an interaction between the genetic and the environmental influences of each station.

### 4.1. Diets and Performance

For all diets, the calculated concentrations of nutrients were confirmed by chemical analyses. Numerical differences between the calculated and the analyzed values were considered within the variable range of analyses and did not interfere with the experimental design. Overall, performance of the animals was at or above the level normally achieved at the respective experimental station using the specific genetics. The performance of animals of treatments 1 and 3 did not differ. As a P deficiency is known to have negative effects on animal performance [22], probably no P deficiency was reached upon the reduction in dietary P in the present study. Vier et al. [22] found effects on animal performance upon a reduction in dietary P. The P level in the study of Vier et al. [22] was higher than the P concentrations in the present study, but the diets did not include phytase and had a wider Ca: available P ratio. Hence, the P availability and thus the overall P utilization was most probably lower than in the present study. Stahly et al. [23] found higher estimated available P needs for lean pigs to optimize FCR and ADG than the diets in the current study supplied. This difference in the response of animals might be explained by the wider Ca:dP ratio in the first feeding phases (2.5 and 2.3:1 vs. 2.1:1) in the diets used in the study of Stahly et al. [23] and different genetic backgrounds of the animals. Because the treatment with the lowest dietary available P concentration had had no effect on animal performance, the diets in the present study were considered to contain adequate dP concentrations to meet the animal requirements. This is supported by the feeding trials conducted by Rieger [24], in which P concentration in the negative control was even lower than in the present experiment, and the positive control—described as a diet to achieve optimal performance—was only slightly higher than in the present experiment. The variation between studies highlights the importance of using available P for feed optimization, taking the Ca:dP ratio into account and adjusting the supplementation of phytase to the age of the animals and the diet composition.

Another reason for differences between studies on P supply of growing pigs might be the protein content in the experimental diets. Lautrou et al. [25] stated that the P requirement of animals is lower if the protein deposition is reduced. Because the diets in the present study were calculated to contain protein concentrations lower than the recommendations of the NRC [18], this might be the reason why no adverse effects of the P reduction were found. Consequently, in the present study, the CP concentration was constant between the dietary treatments within one feeding phase to avoid interaction effects. Under practical conditions, the N concentration also needs to be considered. Assuming the theory of Lautrou et al. [25] applies to the present experiment, a reduction in CP would have allowed an even stronger reduction in dietary P.

However, during the piglet period, animals receiving T2 had significantly lower ADG and higher FCR than animals receiving T1 or T3. This effect was observed at all experimental stations. Because the feed was the only factor all stations had in common, other variables than the diets can be excluded as reasons for the impaired performance. None of the analyzed nutrients give an explanation why animals receiving T2 performed less favorably than animals of T1 and T3. The components that were used for the rations were identical between the treatments within one feeding phase, which is why the diet composition can also be discarded as reason for the differences in the performance of the piglets. A possible explanation for the observed differences in performance might be a contamination of the feeds by mycotoxins, which were not evenly distributed within the batches and thus between the treatments. This could also explain why animals at station 3 showed a stronger reduction in performance by T2 than animals at other stations. Based on the recorded data, it cannot finally be concluded what led to the decrease in animal performance for T2. Because animals receiving T3 had a similar performance than animals receiving T1, the available P level can be excluded as a possible reason. As all feed ingredients were cleaned two times before production of the rations, a contamination, e.g., by mycotoxins, seems quite unlikely, but is the most probable reason for the lower performance when T2 was fed. In order to find an explanation for the differences between T2 and the other treatments, a follow-up piglet feeding experiment was conducted at experimental station 1. The animals received diets calculated to contain identical nutrients and raw materials as diets in the present study. In this experiment, performance between the dietary groups was similar. This underlines the complexity of how feed and management can influence animal performance and animal health. It also demonstrates that under practical conditions a decrease in performance can be caused by a multitude of factors and is not necessarily related to a reduced P concentration in the feeds.

### 4.2. Blood Data, Bone Ash Measures and Mineral Transporters

Collected data on blood serum concentration of inorganic P and Ca were at the known physiological level for all feeding groups [26,27,28]. Accordingly, the measured gene expressions of all currently known Na-P-cotransporters in the kidney cortex were at a physiological level [21]. The XA concentration in fat-free DM of the sampled metatarsi (overall mean: 577 ± 25 g XA/kg fat-free DM, n = 18 animals per dietary treatment) was similar to the values described in the literature for *os metatarsale* III ([24]; 614 ± 3 g XA/kg fat-free DM, n = 4). Expressed on a basis of fat-free DM, P and Ca concentration in the metatarsal bones are again in the same range as the results for *os metatarsale* III of Rieger [24]. In the present study, 106 ± 6 g P/kg ff DM and 205 ±9 g Ca/kg ff DM were found, whilst Rieger [24] described values of 108 ± 3 g P/kg fat-free DM and 222 ± 5 g Ca/kg fat-free DM. It must be considered that Rieger [24] sampled the bones on day 82, and in the present study, the animals were significantly older (approximately 150 d). Age effects on bone mineralization might therefore account for the differences between the studies. Femur characteristics were unaffected by dietary P supply. In turn, diets with pronounced reductions in mineral P levels showed lowered femur mineralization, which was associated with lower trabecular thickness and bone breaking strength [29]. The results of the current study do not indicate a systematic P deficiency in the fattening pigs. However, variabilities in ash contents of vertebral bodies suggest genetic or management components on nutrient allocation [27]. To map P supply in pigs, physiological indicators are used to monitor the interaction of individual organs for balanced mineral homeostasis and thus tissue integrity. Because the data on bone ash of sampled bones, blood parameters and expression of the P transporter in the kidney did not indicate endogenous mechanisms to compensate for any P reductions due to the diets [29,30], it is assumed that all dietary treatments of the current study sufficiently supplied P. One possible explanation for the uniform performance and mineralization might be a more efficient intestinal phytate degradation and an upregulation of the absorption of P in animals receiving diets with lower P concentration [31]. On the other hand, all diets were calculated to meet or exceed the recommendations of the German society of nutrition for dP, which is derived by applying a factorial approach. This means that the potential of the endogenous regulatory mechanism is not yet considered in the recommendations [17], which could allow further adjustment of dietary P levels. On the other hand, this demonstrates that the animals in the present experiment were sufficiently supplied with dP.

Bone ash and kidneys were sampled only at some of the involved experimental stations, due to logistical restrictions. Animals at experimental station 3 showed the strongest reaction on dietary treatment T2, thus possible influences of the dietary treatment on bone ash measures, blood parameters and mineral transporters would have been more likely in samples from station 3 than at the other stations. Because all tissues were at least sampled at location 3, there were most likely no differences at the other stations.

## 5. Conclusions

From the results, it can be concluded that the feed types used in the present study contained sufficient dP to fulfill the requirements of pigs from weaning to slaughter for optimal growth. This indicates that the applied P concentrations in the piglet, grower and finisher diets, which were adjusted according to values described by the German agricultural society [16], are more than adequate to ensure optimal bone mineralization and growth performance. By implication, this means that the P supply under typical German practical recommendations is not accountable for skeletal malformations sometimes reported on farms. The observed constant performance of the animals with a reduced intake at the same time indicates that a further significant reduction of P emissions can be achieved, which contributes to more sustainable animal husbandry and meat production.

## Figures and Tables

**Table 1:** Planned number of animals and number of repetitions for feed uptake at the experimental stations. Total number of piglets at the beginning of the experiment: 695, total number of fattening pigs at the beginning of the fattening phases: 582 (after losses and sacrificed animals during the piglet and fattening phases, total number of animals: 532).

Station	Animals per Repetition		Repetition per Treatment		Feed Uptake on Basis of…	
	Piglets	Fattening Pigs	Piglets	Fattening Pigs	Piglets	Fattening Pigs
1	10	1	12	60	Group	Animal
2	8	1	10	50	Group	Animal
3	1 ^1^	1	24/47 ^2^	60	Animal	Animal
4	-	1	-	24	-	Animal

^1^ station 3: piglets were kept in groups of 12 or 11 animals; ^2^ 24 animals for treatment 1 and 2, 47 animals for treatment 3.

**Table 2:** Composition (%), calculated concentration of selected nutrients (%, unless stated otherwise) and energy of diets for piglets (on 88% DM basis). Phosphorus concentration of the diets declines from T1 to T3.

	Phase P1			Phase P2		
Body Weight	8 to 15 kg			15 to 28 kg		
Treatment	T1	T2	T3	T1	T2	T3
Barley	30.00			29.00		
Wheat	8.00			24.75		
Corn	12.00			15.00		
Corn, expanded	18.00			3.00		
Whey fat concentrate	5.00			1.00		
Soy protein concentrate	6.50			3.00		
Soybean meal	6.40			15.20		
Soybean hulls	2.00			1.35		
Potato protein	5.00			1.00		
Oil	1.50			1.35		
Calciumformiate	1.00			0.50		
Calciumcarbonate	1.000	0.88	0.85	1.30	1.15	1.10
Monocalciumphosphate	0.950	0.70	0.65	0.83	0.61	0.50
Natriumchloride	0.55			0.60		
L-Lysine-HCL	0.50			0.55		
DL-Methionine	0.125			0.13		
Threonine	0.167			0.22		
L-Tryptophane	0.167			0.043		
L-Valine	0.01			0.057		
Organic acid premix	0.85			0.75		
Vitamin Premix	0.398			0.365		
CP	18.00			17.50		
Ca	0.9	0.83	0.81	0.85	0.76	0.73
P	0.55	0.51	0.50	0.53	0.48	0.46
dP	0.41	0.38	0.37	0.39	0.38	0.33
InsP_6_-P	0.16			0.21		
ME (MJ/kg)	13.8			13.5		
Phytase (FTU/kg)	1000					

CP = crude protein, Ca = calcium, P = phosphorus, dP = digestible phosphorus, InsP6-P = phytate bound P, ME = metabolizable energy.

**Table 3:** Composition (%), calculated concentration of selected nutrients (%, unless stated otherwise) and energy of diets for fattening pigs (on 88% DM basis). Phosphorus concentration of the diets declines from T1 to T3.

	Phase F1			Phase F2			Phase F3		
Body Weight	28 to 60 kg			60 to 90 kg			90 to 120 kg		
Treatment	T1	T2	T3	T1	T2	T3	T1	T2	T3
Barley	30.0			29.0			29.0		
Wheat	24.5			24.3			25.0		
Rye	10.0			14.5			16.5		
Corn	10.0			12.0			13.8		
Soybean meal HP	20.0			14.5			11.4		
Soybean hulls	1.000			1.000			1.000		
Oil	0.5			0.5			0.5		
Calciumcarbonate	1.450	1.300	1.250	1.350	1.200	1.050	1.200	1.150	0.900
Monocalciumphosphate	0.700	0.400	0.350	0.500	0.300	0.100	0.450	0.350	0.100
Natriumchloride	0.450			0.450			0.450		
L-Lysine HCl	0.483			0.330			0.317		
DL-Methionine	0.123			0.033			0.033		
Threonine	0.193			0.117			0.120		
L-Tryptophane	0.023			-			-		
L-Valine	0.007			-			-		
Vitamin Premix	0.571			0.520			0.230		
XP	17.50			15.50			14.00		
Ca	0.80	0.68	0.66	0.70	0.62	0.53	0.64	0.60	0.46
P	0.50	0.43	0.42	0.44	0.40	0.35	0.42	0.40	0.33
dP	0.37	0.31	0.30	0.32	0.27	0.24	0.30	0.27	0.21
InsP_6_-P	0.25			0.24			0.23		
ME (MJ/kg)	13.4								
Phytase (FTU/kg)	750			500			300		

**Table 4:** Body weight and average daily weight gain (ADG) of animals during the piglet phases P1 (8–15 kg BW), P2 (15–28 kg BW) and over the complete piglet period (for information on the number of repetitions, please refer to Table 1).

			Body Weight (kg)			ADG (g/d)		
Station	Treatment	Sex	Start P1	End P1	End P2	P1	P2	P1 + P2
1	T1		8.2	13.9	28.3	287	657 ^c^	474 ^c^
		F	8.2	13.5	27.3	270	628	453
		B	8.3	14.2	29.3	303	765	496
	T2		8.3	13.0	26.8	239	620 ^d^	436 ^d^
		F	8.3	13.0	26.5	242	476	427
		B	8.3	13.0	27.1	235	689	440
	T3		8.3	14.1	28.2	292	645 ^cd^	472 ^c^
		F	8.2	14.1	28.0	291	663	469
		B	8.3	14.1	28.3	293	651	476
2	T1		8.9	16.8	34.0	382	757 ^a^	571 ^a^
		F	8.9	16.6	33.4	372	742	560
		B	8.9	16.9	34.6	393	772	582
	T2		8.9	16.4	32.4	360	693 ^b^	529 ^b^
		F	8.9	16.3	32.2	358	697	529
		B	8.8	16.5	32.6	362	689	528
	T3		8.9	16.8	34.1	383	758 ^a^	572 ^a^
		F	9.0	16.8	33.9	379	750	565
		B	8.8	16.8	34.3	388	766	579
3	T1		9.1	20.6	30.8	441	711 ^ab^	536 ^ab^
		F	9.1	20.0	29.5	419	658	503
		B	9.1	21.2	32.0	463	765	569
	T2		9.1	18.5	26.0	361	510 ^e^	413 ^d^
		F	9.0	18.5	25.7	375	476	398
		B	9.2	18.5	26.3	365	545	428
	T3		9.1	20.4	30.0	428	663 ^bc^	508 ^b^
		F	9.0	20.1	29.7	428	663	510
		B	9.0	20.6	30.3	428	662	507
pooledSEM (station × treatment)			0.15	0.26	0.44	8.31	11.70	8.86
Main effects								
1			8.3 ^b^	13.6 ^c^	26.8 ^c^	370 ^c^	641	449
2			8.9 ^a^	16.7 ^b^	33.0 ^a^	320 ^c^	736	557
3			9.1 ^a^	19.8 ^a^	28.5 ^b^	368 ^a^	628	486
	T1		8.7	17.1 ^a^	31.0 ^a^	370 ^a^	708	520
	T2		8.8	15.9 ^b^	28.4 ^b^	320 ^b^	608	459
	T3		8.8	17.0 ^a^	30.8 ^a^	368 ^a^	688	514
		F	8.7	16.5	29.6 ^b^	346	650	490
		B	8.8	16.8	30.5 ^a^	359	686	511
ANOVA *p*-values								
Treatment			0.960	<0.001	< 0.001	<0.001	<0.001	<0.001
Sex			0.838	0.164	0.010	0.065	0.003	0.005
Station			<0.001	<0.001	< 0.001	<0.001	<0.001	<0.001
Sex × Treatment			0.996	0.398	0.181	0.902	0.066	0.103
Station × Treatment			0.997	0.245	0.160	0.102	<0.001	0.026
Station × Sex			0.941	0.900	0.819	0.902	0.215	0.616
Station × Sex × Treatment			0.888	0.968	0.973	0.925	0.519	0.749

^a, b, c, d, e^ Values with different superscripts differ significantly at a significance level of α = 0.05; F = female, B = barrow.

**Table 5:** Body weight (BW) and average daily weight gain (ADG) of animals during the fattening phases F1 (28–60 kg BW), F2 (60–90 kg BW), F3 (90–120 kg BW) and over the complete fattening phase; for information on the number of repetitions, please refer to Table 1.

			BW (kg) at the Beginning of Fattening Phase			Final BW (kg)	ADG (g/d)			
Station *	Treatment	Sex	F1	F2	F3		F1	F2	F3	F1–F3
1	T1		28.2 ^d^	60.5 ^bcd^	90.4 ^c^	124.1	940	1052	1077	1012
		F	26.7	60.0	90.8	123.9	882 ^hij^	1005	1036	964
		B	29.8	61.0	89.9	124.2	999 ^cde^	1010	117	1061
	T2		25.8 ^e^	60.0 ^bcd^	90.2 ^c^	123.5	915	1078	1077	1011
		F	24.9	60.3	90.5	124.0	893 ^hi^	1072	1084	1000
		B	26.7	59.6	89.9	123.0	938 ^ghf^	1084	1070	1023
	T3		27.8 ^d^	60.2 ^bcd^	90.1 ^c^	124.0	927	1086	1069	1016
		F	28.6	59.8	90.2	123.8	884 ^hij^	1032	1041	976
		B	24.9	60.6	90.0	124.1	970 ^efg^	1140	1097	1056
2	T1		33.4 ^b^	64.0 ^a^	94.0 ^b^	121.0	936	959	948	949
		F	33.0	62.5	90.6	119.2	900 ^hi^	906	930	909
		B	33.8	65.6	97.5	122.9	972 ^defg^	1012	965	989
	T2		32.8 ^bc^	64.8 ^a^	96.5 ^a^	121.2	988	998	974	984
		F	32.7	63.0	92.3	120.0	932 ^fgh^	935	895	919
		B	32.8	66.7	100.8	122.4	1044 ^abc^	1062	1053	1049
	T3		33.3 ^b^	64.3 ^a^	95.9 ^a^	121.8	952	1002	1045	990
		F	33.7	63.7	93.6	121.0	923 ^gh^	950	995	950
		B	33.0	64.9	98.1	122.5	980 ^cdef^	1054	1095	1030
3	T1		36.5 ^a^	61.6 ^bc^	96.8 ^a^	121.8	897	1004	802	904
		F	34.5	57.4	88.9	116.9	818 ^j^	900	756	821
		B	38.6	65.9	104.7	126.7	976 ^cdefg^	1108	849	986
	T2		31.0 ^c^	57.8 ^e^	92.2 ^bc^	118.8	956	983	857	935
		F	30.0	53.9	85.6	114.8	854 ^ij^	907	789	850
		B	32.1	61.7	98.7	122.8	1058 ^abc^	1059	926	1020
	T3		36.4 ^a^	62.0 ^b^	97.2 ^a^	121.5	913	1007	869	935
		F	35.3	59.8	91.9	119.3	875 ^hij^	917	808	865
		B	37.6	64.2	102.6	123.7	950 ^efgh^	1097	931	1005
4	T1		27.5 ^d^	59.9 ^cd^	90.3 ^c^	123.1	1057	1093	1211	1110
		F	26.6	57.4	89.7	122.3	1052 ^abc^	1031	1167	1072
		B	28.3	65.9	90.9	123.9	1063 ^ab^	1108	1255	1147
	T2		26.0 ^e^	59.5 ^de^	89.9 ^c^	122.5	1042	1087	1169	1089
		F	26.3	53.9	89.7	121.9	1034 ^bc^	1062	1128	1065
		B	25.7	61.2	90.1	123.	1049 ^abc^	1059	1209	1113
	T3		27.4 ^d^	59.7 ^d^	90.5 ^c^	123.4	1054	1105	1177	1104
		F	27.0	59.5	90.2	122.6	1023 ^bcd^	1055	1111	1058
		B	27.7	64.2	90.7	124.3	1085 ^a^	1097	1243	1151
Pooled SEM (station × treatment)			0.51	0.50	0.66	0.52	13.7	15.9	22.5	12.3
Main effects										
1			27.3	60.2	90.2	123.9	927	1072 ^a^	1074 ^b^	1013
2			33.2	64.4	95.5	121.3	959	986 ^b^	989 ^c^	974
3			34.6	60.5	95.4	120.7	952	988 ^b^	843 ^d^	925
4			27.0	59.7	90.2	123.0	1051	1095 ^a^	1185 ^a^	1101
	T1		31.4	61.5	92.9	122.5 ^a^	958	1027	1010	994
	T2		28.9	60.5	92.2	121.5 ^b^	975	1037	1019	1005
	T3		31.2	61.6	93.4	122.7 ^a^	962	1050	1040	1011
		F	29.8	59.9	90.3	120.8	922	981	978	954
		B	31.2	62.4	95.3	123.6	1007	1095	1067	1046
ANOVA *p*-values										
Treatment			<0.001	0.012	0.067	0.010	0.239	0.189	0.243	0.181
Sex			<0.001	0.001	<0.001	<0.001	<0.001	<0.001	<0.001	<0.001
Station			<0.001	<0.001	<0.001	<0.001	<0.001	<0.001	<0.001	<0.001
Sex × Treatment			0.061	0.181	0.163	0.073	0.495	0.137	0.764	0.840
Station × Treatment			0.005	0.006	0.001	0.389	0.032	0.716	0.059	0.245
Station × Sex ^+^			0.034	<0.001	<0.001	<0.001	<0.001	0.010	0.159	0.002
Station × Sex × Treatment			0.855	0.188	0.416	0.355	0.035	0.515	0.351	0.122

* Station includes the effect of the experimental station, including the used genetics; ^+^ ls-means are not displayed in favor of readability; F = female, B = barrow; ^a, b, c, d, e, f, g, h, i, j^ values with different superscripts differ significantly at a significance level of α = 0.05.

**Table 6:** Feed consumption and feed conversion ratio (FCR) during piglet phases P1 (8–15 kg BW), P2 (15–28 kg BW) and over the complete piglet period (for information on the number of repetitions, please refer to Table 1).

		Feed Consumption (g/animal/d)			FCR (kg Feed/kg Weight Gain)		
Station *	Treatment	P1	P2	P1 + P2	P1	P2	P1 + P2
1	T1	351	965	661	1.25	1.48 ^c^	1.41 ^cde^
	T2	331	924	625	1.37	1.49 ^c^	1.45 ^cd^
	T3	359	954	660	1.23	1.49 ^bc^	1.41 ^cde^
2	T1	506	1147	834	1.35	1.52 ^bc^	1.46 ^cd^
	T2	527	1130	830	1.45	1.65 ^b^	1.58 ^ab^
	T3	524	1158	845	1.41	1.53 ^bc^	1.49 ^bc^
3	T1	552	1079	736	1.26	1.53 ^bc^	1.37 ^e^
	T2	488	966	655	1.36	1.92 ^a^	1.60 ^a^
	T3	529	1050	711	1.25	1.61 ^b^	1.40 ^de^
Pooled SEM (station × treatment)		15.3	26.4	17.96	0.03	0.04	0.02
Main effects							
1		347 ^b^	948 ^c^	625 ^c^	1.28 ^b^	1.49	1.42
2		519 ^a^	1145 ^a^	837 ^a^	1.40 ^a^	1.56	1.51
3		523 ^a^	1031 ^b^	701 ^b^	1.29 ^b^	1.69	1.46
	T1	470	1064	732	1.29 ^b^	1.51	1.42
	T2	448	1006	700	1.40 ^a^	1.68	1.54
	T3	471	1054	729	1.30 ^b^	1.54	1.43
ANOVA *p*-values							
Treatment		0.325	0.118	0.092	<0.001	<0.001	<0.001
Station		<0.001	<0.001	<0.001	<0.001	<0.001	0.002
Treatment × Station		0.335	0.645	0.576	0.782	<0.001	0.001

* Station includes the effect of the experimental station, including the used genetics ^a, b, c, d, e^ Values with different superscripts differ significantly at a significance level of α = 0.05.

**Table 7:** Feed consumption and feed conversion ratio (FCR) during the three fattening phases F1 (28–60 kg BW), F2 (60–90 kg BW), F3 (90–120 kg BW) and over the complete fattening phase (for information on the number of repetitions, please refer to Table 1).

			Feed Consumption (kg/animal/d)				FCR (kg feed/kg Weight Gain)			
Station	Treatment		F1	F2	F3	F1–F3	F1	F2	F3	F1–F3
1	T1		1.97	2.63	2.99	2.50	2.10	2.52 ^cd^	2.82	2.48
	T2		1.88	2.61	3.04	2.46	2.05	2.43 ^de^	2.86	2.44
	T3		1.94	2.69	2.99	2.51	2.10	2.48 ^cde^	2.82	2.46
2	T1		1.92	2.52	2.85	2.41	2.06	2.63 ^ab^	3.04	2.54
	T2		1.93	2.48	2.87	2.39	1.95	2.50 ^cde^	3.08	2.44
	T3		1.95	2.64	2.99	2.49	2.05	2.64 ^ab^	2.94	2.51
3	T1		1.72	2.41	2.77	2.33	1.92	2.39 ^de^	3.54	2.59
	T2		1.70	2.33	2.76	2.28	1.77	2.37 ^e^	3.23	2.44
	T3		1.73	2.50	2.83	2.36	1.90	2.47 ^cde^	3.26	2.52
4	T1		1.98	2.82	3.14	2.61	1.87	2.59 ^abc^	2.61	2.35
	T2		1.94	2.87	3.15	2.61	1.86	2.65 ^a^	2.71	2.40
	T3		1.94	2.81	3.15	2.61	1.84	2.55 ^bc^	2.69	2.36
Pooled SEM (station × treatment)			0.028	0.041	0.054	0.032	0.023	0.037	0.059	0.027
Main effects										
1			1.93	2.64	3.01	2.49	2.09 ^a^	2.48	2.83 ^c^	2.46 ^a^
2			1.93	2.55	2.90	2.43	2.02 ^b^	2.59	3.02 ^b^	2.50 ^a^
3			1.72	2.41	2.79	2.32	1.86 ^c^	2.41	3.34 ^a^	2.52 ^a^
4			1.95	2.83	3.15	2.61	1.86 ^c^	2.60	2.67 ^d^	2.37 ^b^
	T1		1.90	2.60	2.94	2.46	1.99 ^a^	2.53	3.00	2.49 ^a^
	T2		1.86	2.57	2.96	2.44	1.91 ^b^	2.49	2.97	2.43 ^b^
	T3		1.89	2.66	2.99	2.49	1.97 ^a^	2.54	2.93	2.46 ^ab^
		F	1.78	2.38	2.75	2.29	1.94 ^b^	2.43	2.88	2.41
		B	1.98	2.85	3.17	2.64	1.98 ^a^	2.61	3.05	2.51
ANOVA *p*-values										
Treatment			0.205	0.026	0.460	0.107	<0.001	0.188	0.263	0.019
Sex			<0.001	<0.001	<0.001	<0.001	0.004	<0.001	<0.001	<0.001
Station			<0.001	<0.001	<0.001	<0.001	<0.001	<0.001	<0.001	<0.001
Sex × Treatment ^+^			0.138	0.237	0.482	0.892	0.434	0.892	0.867	0.872
Station × Treatment			0,435	0.142	0.728	0.734	0.060	0.023	0.094	0.062
Station × Sex ^+^			< 0.001	0.004	0.053	0.021	0.690	0.009	0.865	0.414
Station × Sex × Treatment ^+^			0.086	0.849	0.613	0.615	0.126	0.595	0.741	0.153

^+^ ls-means are not displayed in favor of readability; F = female, B = barrow; ^a, b, c, d, e^ Values with different superscripts differ significantly at a significance level of α = 0.05.

**Table 8:** Serum concentration of phosphorous (P) and calcium (Ca) in fattening pigs fed with declining P and Ca concentrations throughout grower and finisher periods (diets 1 to 3). Samples were derived from station 3 (n = 40 animals).

Treatment	Sex	P (mmol/L)	Ca (mmol/L)
T1	F	3.2 ^ab^	2.6
	B	3.1 ^ab^	2.6
T2	F	3.1 ^ab^	2.6
	B	3.3 ^a^	2.7
T3	F	3.2 ^ab^	2.7
	B	2.9 ^b^	2.6
Pooled SEM (treatment × sex)		0.35	0.17
Main effects			
T1		3.1	2.6
T2		3.2	2.7
T3		3.1	2.7
	F	3.2	2.6
	B	3.1	2.6
ANOVA *p*-values			
Treatment		0.637	0.886
Sex		0.856	0.981
Treatment × Sex		0.040	0.580

^a, b^ Values with different superscripts differ significantly at a significance level of α = 0.05; F = female, B = barrow.

**Table 9:** Expression (log2-transformed) of genes encoding Na-/P-co-transporters in kidney cortex of fattening pigs fed diets with declining P and Ca concentrations throughout grower and finisher periods (diets 1 to 3). Samples were retrieved from station 3 (n = 40 animals).

		SLC17			SLC20			SLC34		
		A1	A2	A3	A4	A1	A2	A1	A2	A3
		(NPT1)	(NPT3)	(NPT4)	(NPT5)	(PiT1)	(PiT2)	(NPT2a)	(NPT2b)	(NPT2c)
Treatment	Sex									
T1	F	15.4	7.3	16.3	9.9	12.2	10.0	19.1	4.9	12.4
	B	15.0	6.6	16.5	8.6	11.4	9.5	18.9	4.5	12.3
T2	F	15.0	6.5	16.5	8.9	11.6	9.8	18.9	4.9	11.5
	B	15.1	6.4	16.5	8.6	11.6	9.8	18.9	4.9	12.2
T3	F	14.9	6.9	16.0	9.8	11.2	9.6	18.8	4.4	12.0
	B	15.2	6.9	16.6	9.0	11.7	9.7	18.7	4.3	12.2
Pooled SEM (treatment × sex)		0.27	0.40	0.36	0.58	0.31	0.21	0.16	0.52	12.1
Main effects										
T1		15.2	7.0	16.4	9.2	11.8	9.8	19.0	4.7	12.3
T2		15.0	6.5	16.5	8.8	11.6	9.8	18.9	4.9	11.9
T3		15.0	6.9	16.3	9.4	11.5	9.6	18.8	4.4	12.1
	F	15.1	6.9	16.3	9.6	11.7	9.8	18.9	4.7	12.0
	B	15.1	6.6	16.5	8.7	11.6	9.6	18.8	4.6	12.2
ANOVA *p*-values										
Treatment		0.750	0.476	0.993	0.533	0.420	0.719	0.300	0.563	0.074
Sex		0.484	0.139	0.684	0.097	0.121	0.333	0.442	0.727	0.100
Treatment × Sex		0.439	0.553	0.987	0.567	0.049	0.508	0.801	0.928	0.203

F = female, B = barrow.

**Table 10:** Weight, dry matter (DM), fat-free DM (ffDM), crude ash (CA) and phosphorus and calcium concentration in metatarsal bones of piglets at the end of the piglet period (body weight approximately 28 kg, n = 24 animals).

		Weight (g)	DM (g)	ff DM (g)	CA (g)	Ca in CA (g/kg)	P in CA (g/kg)	Ca (g)	P (g)
Station	Treatment								
2	T1	9.8 ^bc^	6.8	4.7 ^bc^	2.7	349.8	176.0	0.9	0.5
	T2	10.2 ^bc^	6.8	4.9 ^bc^	2.7	350.0	177.5	1.0	0.5
	T3	9.1 ^c^	6.4	4.4 ^c^	2.5	350.8	178.0	0.9	0.4
3	T1	12.2 ^a^	8.4	5.6 ^a^	3.2	343.0	179.8	1.1	0.6
	T2	10.8 ^b^	7.6	4.8 ^bc^	2.8	343.3	172.5	1.0	0.6
	T3	12.1 ^a^	7.7	5.1 ^ab^	2.8	346.5	175.8	1.0	0.6
Pooled SEM (station × treatment)		0.42	0.29	0.18	0.10	1.50	2.37	0.04	0.02
Main effects									
2		9.7	6.7 ^b^	4.7	2.6 ^b^	350.2 ^a^	177.2	0.9 ^b^	0.5 ^b^
3		11.7	7.9 ^a^	5.2	2.9 ^a^	344.3 ^b^	176.0	1.0 ^a^	0.6 ^a^
	T1	11.0	7.6	5.2	2.9	346.4	177.9	1.0	0.5
	T2	10.5	7.2	4.7	2.8	346.6	175.0	1.0	0.5
	T3	10.6	7.1	4.7	2.7	348.6	176.9	0.9	0.5
ANOVA *p*-values									
Treatment		0.436	0.164	0.049	0.061	0.282	0.484	0.095	0.372
Station		<0.001	<0.001	0.002	0.004	<0.001	0.555	0.007	<0.001
Treatment × Station		0.033	0.461	0.038	0.173	0.635	0.198	0.163	0.309

^a, b, c^ Values with different superscripts differ significantly at a significance level of α = 0.05; F = female, B = barrow.

**Table 11:** Femur length and weight (n = 76 animals) and crude ash (CA) of the left femur (n = 76 animals) and vertebral bodies (n = 40 animals) of female pigs and barrows at the end of the fattening period. Diets contained declining P and Ca concentrations (diets 1 to 3).

			Femur			Vertebral Bodies
Station	Treatment	Sex	Length	Weight	CA	CA
			(cm)	(g)	(%)	(%)
2	T1		20.2	424.6	22.6	18.1 ^b^
		F	20.3	421.2	22.7 ^a^	17.4
		B	20.1	428.0	22.5 ^a^	18.8
	T2		20.3	423.6	21.6	20.2 ^a^
		F	20.5	433.4	20.6 ^ab^	20.8
		B	20.1	413.8	22.6 ^a^	19.7
	T3		20.0	398.2	22.1	16.0 ^b^
		F	20.1	391.8	21.8 ^ab^	16.3
		B	19.8	404.5	22.3 ^a^	15.7
3	T1		20.5	437.4	21.8	21.3 ^a^
		F	20.8	447.3	22.7 ^a^	21.8
		B	20.1	427.5	21.0 ^ab^	20.8
	T2		20.5	466.1	22.0	21.5 ^a^
		F	20.3	465.4	22.5 ^a^	20.4
		B	20.6	466.8	21.6 ^ab^	22.6
	T3		20.6	447.6	21.4	21.8 ^a^
		F	20.8	453.8	20.5 ^b^	21.9
		B	20.4	441.3	22.2 ^a^	21.7
Pooled SEM (station × treatment × sex)			0.24	13.29	0.586	0.951
Main effects						
2			20.2 ^b^	415.5 ^b^	22.1	18.1
3			20.5 ^a^	450.4 ^a^	21.7	21.6
	T1		20.3	431.0	22.2	19.7
	T2		20.4	444.8	21.8	20.9
	T3		20.3	422.9	21.7	18.9
	F		20.4	435.5	21.8	19.8
	B		20.2	430.3	22.0	19.9
ANOVA *p*-values						
Treatment			0.811	0.070	0.429	0.019
Sex			0.086	0.509	0.451	0.846
Station			0.017	<0.001	0.331	<0.001
Sex × Treatment			0.458	0.876	0.055	0.790
Station × Treatment			0.379	0.129	0.312	0.006
Station × Sex			0.934	0.510	0.127	0.702
Station × Sex × Treatment			0.258	0.387	0.049	0.120

^a, b^ Values with different superscripts differ significantly at a significance level of α = 0.05; F = female, B = barrow.

## Data Availability

Raw data of this study are stored at each research station and centrally at the servers of the Agricultural Chamber of North Rhine Westphalia. Additionally, all SAS codes and generated outputs are digitally stored at the servers of the Agricultural Chamber of North Rhine Westphalia.

## Supplementary Materials

The following supporting information can be downloaded at https://www.mdpi.com/article/10.3390/ani13111774/s1, Table S1: Analyzed nutrient composition of the experimental diets during piglet phases 1 and 2 (88% DM); Table S2: Analyzed nutrient composition of the experimental diets during fattening phases F1 to F3 (88% DM).
