# Supplementary material for: Stepwise Reduction of Dietary Phosphorus in Diets for Piglets and Fattening Pigs of Different Genetic Origin Housed under Various Station Environments—A Ringtest
Source: Animals (Basel). 2023 May 26;13(11):1774. doi: 10.3390/ani13111774 (PMC10251985; doi:10.3390/ani13111774)
Supplement: Supplementary file 1 [file animals-13-01774-s001.zip › animals-2376712-supplementary.pdf]

Table S1. Analyzed nutrient composition of the experimental diets during piglet phases 1 and 2 (88% DM).

		Phase P1			Phase P2		
	**Body Weight**	**8 to 15 kg**			**15 to 28 kg**		
	**Treatment**	**T1**	**T2**	**T1**	**T1**	**T2**	**T1**
Ash	(%)	6.01	5.18	5.18	5.64	5.45	5.30
CP	(%)	18.46	17.58	18.50	18.00	17.70	17.50
CF	(%)	3.55	3.32	3.24	3.36	3.50	3.50
EE	(%)	4.1	5.60	5.31	4.65	4.26	4.50
XS	(%)	40.73	40.46	40.31	41.82	42.50	42.20
Lys	(%)	1.38	1.14	1.22	1.22	1.11	1.25
Met	(%)	0.36	0.31	0.32	0.31	0.36	0.30
Thr	(%)	0.87	0.78	0.81	0.78	0.85	0.80
Val	(%)	0.89	0.79	0.78	0.79	0.82	0.80
Ca	(%)	0.90	0.83	0.75	0.91	0.83	0.81
P	(%)	0.53	0.52	0.51	0.56	0.53	0.50
ME	(MJ/kg)					13.58	13.60
AD-	(g/kg)	43.36	46.16	51.13	k.A.	k.A.	k.A.
aND-	(g/kg)	116.28	130.63	133.72	k.A.	k.A.	k.A.
Phytase	(FTU/kg)	1300	1300	1390	1060	1390	1500

Table S2. Analyzed nutrient composition of the experimental diets during fattening phases F1 to F3 (88% DM).

		Phase F1			Phase F2			Phase F3		
	**Body Weight**	**28 to 60 kg**			**60 to 90 kg**			**90 to 120 kg**		
	**Treatment**	**T1**	**T2**	**T3**	**T1**	**T2**	**T3**	**T1**	**T2**	**T3**
Ash	(%)	5.12	4.89	4.8	4.49	4.26	4.06	4.32	4.24	3.74
CP	(%)	17.48	17.86	17.5	15.96	15.76	15.06	14.58	14.25	14.35
XL	(%)	2.41	2.39	2.4	3.49	3.37	2.77	2.82	2.93	3.13
XF	(%)	3.32	3.39	3.2	3.09	3.27	3.37	3.52	3.84	3.44
Starch	(%)	44.20	43.50	44.1	47.19	46.87	46.08	47.57	47.99	49.51
Lys	(%)	1.20	1.27	1.2	0.95	0.92	0.97	0.87	0.98	0.89
Met	(%)	0.35	0.32	0.3	0.239	0.25	0.24	0.23	0.26	0.26
Val	(%)	0.78	0.81	0.8	0.65	0.67	0.68	0.66	0.71	0.63
Thr	(%)	0.81	0.82	0.78	0.67	0.63	0.64	0.61	0.66	0.64
Na	(%)	0.20	0.25	0.3	0.21	0.208	0.22	0.22	0.23	0.21
Ca	(%)	0.77	0.72	0.7	0.72	0.63	0.58	0.68	0.70	0.49
P	(%)	0.53	0.46	0.4	0.46	0.41	0.37	0.44	0.41	0.34
ME	(MJ/kg)	13.36	13.37	13.4	13.67	13.58	13.28	13.28	13.24	13.54
AD-Fom	(g/kg)	4.02	4.89	4.5	4.59	4.56	4.56	4.32	4.55	5.05
aND-Fom	(g/kg)	10.35	9.88	10.3	10.18	10.8	11.40	11.06	10.81	11.32
